# Dietary L-Carnitine Affects Leukocyte Count and Function in Dairy Cows Around Parturition

**DOI:** 10.3389/fimmu.2022.784046

**Published:** 2022-03-16

**Authors:** Susanne Ursula Kononov, Jennifer Meyer, Jana Frahm, Susanne Kersten, Jeannette Kluess, Susanne Bühler, Anja Wegerich, Jürgen Rehage, Ulrich Meyer, Korinna Huber, Sven Dänicke

**Affiliations:** ^1^ Institute of Animal Nutrition, Friedrich-Loeffler-Institut, Federal Research Institute for Animal Health, Braunschweig, Germany; ^2^ Department of Functional Anatomy of Livestock, Institute of Animal Science, University of Hohenheim, Stuttgart, Germany; ^3^ Clinic for Cattle, University of Veterinary Medicine Hannover, Foundation, Hannover, Germany

**Keywords:** L-carnitine, dairy cow, parturition, phagocytosis, ROS production, leukocyte functionality

## Abstract

In early lactation, an energy deficit leading to a negative energy balance (NEB) is associated with increased susceptibility to disease and has been shown to be an important factor during transition in dairy cows. L-carnitine as a key factor in the mitochondrial transport of fatty acids and subsequently for β-oxidation and energy release is known to modulate mitochondrial biogenesis and thus influence metabolism and immune system. In the current study, we characterized hematological changes around parturition and investigated the potential effects of dietary L-carnitine supplementation on immune cell functions. For this approach, dairy cows were assigned either to a control (CON, n = 30) or an L-carnitine group [CAR, n = 29, 25 g rumen-protected L-carnitine per cow and day (d)]. Blood samples were taken from d 42 *ante partum* (*ap*) until d 110 *post-partum* (*pp*), with special focus and frequent sampling from 0.5 to72 h post-calving to clarify the impact of L-carnitine supplementation on leukocyte count, formation of reactive oxygen species (ROS) in polymorphonuclear cells (PMN) and peripheral mononuclear cells (PBMC) and their phagocytosis activity. Blood cortisol concentration and the capacity of PBMC proliferation was also investigated. All populations of leukocytes were changed during the peripartal period, especially granulocytes showed a characteristic increase up to 4 h *pp*. L-carnitine supplementation resulted in increased levels of eosinophils which was particularly pronounced one day before to 4 h *pp*, indicating a possible enhanced support for tissue repair and recovery. Non-supplemented cows showed a higher phagocytic activity in PBMC as well as a higher phagocytic capacity of PMN during the most demanding period around parturition, which may relate to a decrease in plasma levels of non-esterified fatty acids reported previously. L-carnitine, on the other hand, led to an increased efficiency to form ROS in stimulated PMN. Finally, a short period around calving proved to be a sensitive period in which L-carnitine administration was effective.

## 1 Introduction

During the transition period dairy cows have to cope with physiological consequences of calving and also with metabolic consequences of a negative energy balance (NEB) due to high energy requirements for beginning milk production and an inadequate feed intake (1–3). Mobilization of body reserves provides energy and is mainly necessary for milk synthesis. However, high-yielding dairy cows develop an increased susceptibility to metabolic disorders and an impaired immune responsiveness during this period which is discussed in the context of individual physiological adaptability (4). In support of energy metabolism, L-carnitine is essential because of its function in transporting cytosolic long-chain fatty acids into the mitochondrial matrix where fatty acid-β-oxidation for generation of ATP occurs (5, 6).

Additionally, in human studies, L-carnitine demonstrated antioxidant functions and modulatory effects on platelet activation (7–9). However, significant effects of L-carnitine have also been demonstrated in cells in which mitochondria are not present. Membrane-stabilizing effects of L-carnitine *via* interaction with cytoskeletal membrane proteins have been known for some time and were investigated mainly in human erythrocytes *in vitro* (10, 11). These membrane-stabilizing effects may also be responsible for higher platelet counts in dairy cows supplemented with L-carnitine (12). At the performance level, dietary L-carnitine supplementation in transition dairy cows was described to increase milk yield in early lactation and also milk fat percentage in the first week of lactation, assuming an improved peripartal energy metabolism (13). In these L-carnitine supplemented cows the export of triglycerides (TG) from liver into blood seemed to be increased during the last 14 days *ante partum* (*ap)*, whereas non-esterified fatty acids (NEFA) tended to be lower in supplemented cows directly before calving compared to non-supplemented cows (13). Other studies reported a decrease in liver lipid accumulation for L-carnitine supplemented dairy cows during the transition period and also in cows infused with L-carnitine abomasally (14, 15) indicating an improved capacity for hepatic fatty acid oxidation. At molecular level, an up-regulation of bovine hepatic genes involved in L-carnitine synthesis and L-carnitine uptake was demonstrated within the first week after calving (5) which indicated a critical time for energy metabolism at a cellular level. Furthermore, in different experiments it could be shown that an L-carnitine supplementation in the transition period of dairy cows supported the energy metabolism, more specifically the lipid metabolism (16). Therefore, it can be hypothesized that insufficient endogenous synthesis of L-carnitine could lead to affected cellular energy metabolism and consequently impaired cellular functions which may compromise immunological processes. Evidence for this can be found in studies describing a suppression of the immune system during the peripartal period (4, 17, 18). Concrete evidence of functional impairment of bovine leukocytes by ketone bodies has been reported (19, 20). Hammon et al. (21) demonstrated a significant but modest correlation between *in vivo* plasma NEFA concentration and neutrophil oxidative burst activity *in vitro*. In addition to the changes in energy metabolism during transition in dairy cows, various studies also confirmed endocrine, hematological and immunological changes (22, 23), although many correlations are not investigated in depth, and it is unclear which are the orchestrating factors for successful adaptation during transition in dairy cows. The presence of stress hormones is often associated with alterations in number and function of circulating blood cells. Physiological stress around parturition triggers the production of these hormones, like glucocorticoids, which are described to modulate cellular responses to stress (24–26) and to alter cellular functions of blood leukocytes (27–30) as well as the number of circulating polymorphonuclear cells (PMN) (31). Blood cell populations, like PMN and lymphocytes are important effectors of the innate as well as the adaptive immune system and are particularly challenged during time of parturition as frequently described for humans (32) but also in some studies on dairy cows (33).

A better understanding of temporal hematologic changes and of the impairment of leukocyte function around parturition may be important to enhance recovery, stabilize immunologic responses, and improve energy metabolism in cows. A large number of studies have been conducted to investigate the capacity of supplemental L-carnitine with, however, largely inconsistent results.

Therefore, the present study was designed to determine the effects of a dietary L-carnitine supplementation on blood leukocyte count and function in pluriparous dairy cows with special focus on the time of parturition. We hypothesized that L-carnitine supplementation would support energy metabolism at cellular level in activated cells and thereby affect blood leukocytes positively during the critical time of parturition.

## 2 Materials and Methods

### 2.1 Experimental Design and Sample Collection

**Figure 1:**
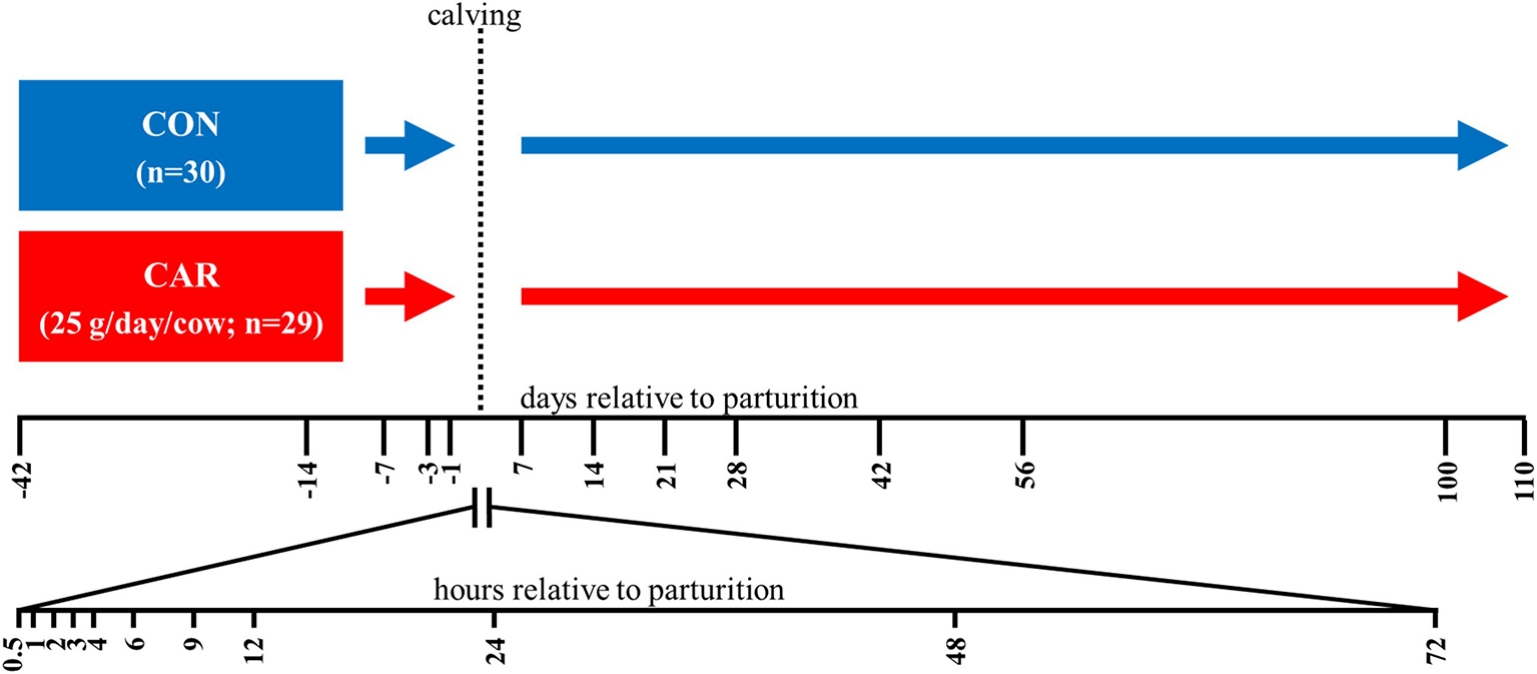
Experimental design. The German Holstein dairy cows were assigned to a control (CON) or an L-carnitine supplemented group (CAR) (Carneon 20 Rumin-Pro, Kaesler Nutrition GmbH, Cuxhaven, Germany). To balance the fat content of the L-carnitine product, CON contained an equivalent fat product (BergaFat F-100 HP, Berg-Schmidt GmbH & Co. KG, Hamburg, Germany) as used for the rumen protection of the L-carnitine. Blood samples were collected from d 42 *ante partum* (*ap)* until d 110 *post-partum* (*pp)* and in a higher frequency during the first 72 h *pp*. The number of cows actually sampled per experimental day and group were listed in the table below.

The experiment was performed at the experimental station of the Institute of Animal Nutrition, Friedrich-Loeffler-Institut (FLI), Braunschweig, Germany in accordance with the German Animal Welfare Act concerning the protection of experimental animals and was approved by Lower Saxony State Office for Consumer Protection and Food Safety (LAVES), Oldenburg, Germany. The entire experiment was described in Meyer et al. (13). From a pool of pluriparous pregnant and healthy German Holstein dairy cows a total of 59 animals was assigned randomly to two feeding groups (with (CAR) or without (CON) a supplementation of rumen-protected L-carnitine) (**Figure 1**) considering an even distribution of body weight (788 ± 220 kg), body condition score (2.5-4.75) and number of lactation (2-5 lactations). The cows in CAR received 25 g per cow and day L-carnitine (Carneon 20 Rumin-Pro, Kaesler Nutrition GmbH, Cuxhaven, Germany), which was included in the concentrate feed. Before parturition until one d *post-partum* (*pp)* the animals received a partial mixed ration (PMR) (20% concentrate and 80% roughage). After parturition, all animals received initially a diet of 30% concentrate and 70% roughage. The concentrate proportion was increased up to 50% of the diet within 14 d and remained at this proportion until the end of the trial. PMR was offered by feed-weigh troughs (RIC, System Insentec B.V., Marknesse, The Netherlands) and the concentrate by concentrate feeding stations (Insentec B.V., Marknesse, The Netherlands) based on dry matter in accordance with the recommendations of the Society of Nutrition Physiology (GfE) for nutrient and energy supply. Water was offered *ad libitum*.

Blood was collected from *Vena jugularis externa*, either through an indwelling catheter in the period from 0.5 h until 12 h *pp* (2.4 mm × 200 mm Teflonkatheter, Walter Veterinär-Instrumente e.K., Baruth/Mark, Germany) or through needle puncture at all other time points into 9 ml tubes containing ethylenediaminetetraacetic acid (EDTA) or heparin and serum tubes. All blood tubes were purchased from Sarstedt AG & Co. KG (Nümbrecht, Germany). Experimental groups, number of animals and sample time points are illustrated in **Figure 1**.

### 2.2 Analyses

#### 2.2.1 Hematology

Hematological determinations were performed immediately after sampling in EDTA whole blood using the automated analyzer Celltac-α (MEK 6450, Nihon Kohden, Qinlab Diagnostik, Weichs, Germany). The white blood cell count included: total white blood cell count (WBC, 10^3^/µl), lymphocytes, granulocytes (including neutrophil and basophil granulocytes), monocytes and eosinophils. Lymphocytes, granulocytes, monocytes and eosinophil were expressed as giga per liter and percentage. The absolute cell counts were used to offset percentages of flow cytometric analyses and thus to calculated absolute numbers of phagocytosing and ROS-producing cells and numbers of cells of specific lymphocyte subsets.

Additionally, blood smears were prepared and air-dried before staining according to Pappenheim. For this, cell smears were stained with a combination of May-Grünwald and Giemsa solution. Differentiation of polymorphonuclear (PMN) leukocytes was performed manually by light microscopy (Eclipse E200, Nikon Instruments Europe b.v., Amsterdam, The Netherlands) based on their morphological and cytochemical staining characteristics. PMN cells were classified as mature cells when the nuclei were divided at least into two morphologically distinct segments (segmented neutrophils) or classified as immature cells when the nuclei appeared band formed (banded neutrophils). At least 100 cells were counted per sample and the conversion to absolute numbers was conducted using results from automated cell counts.

#### 2.2.2 Phagocytosis

**Figure 2:**
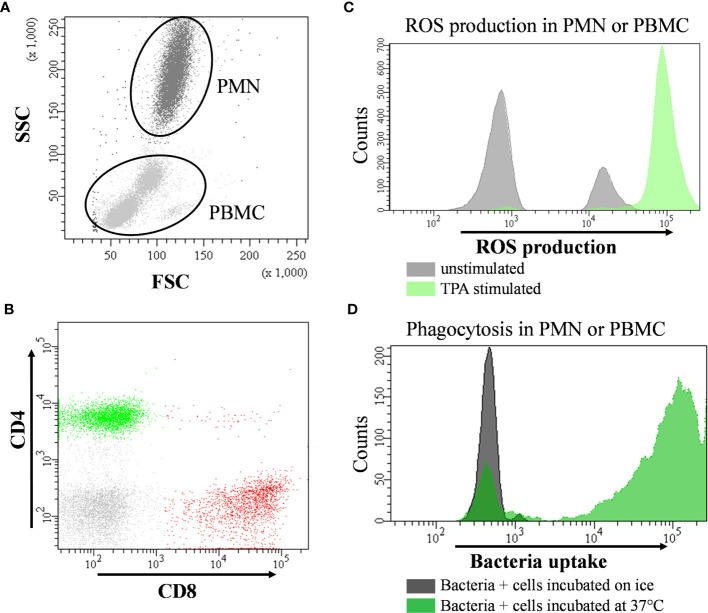
Representative flow cytometry images and hierarchical gating strategies for leukocyte subsets in bovine peripheral blood. **(A)** for the phagocytosis assay cells were first gated for peripheral blood mononuclear cells (PBMC) and polymorphonuclear cells (PMN) according to their morphological properties using forward (F-) and side (S-) scatter (-SC) settings. To distinguish cells from bacteria, DNA content was used for definition (small histogram). Percentage of phagocytosing PBMC or PMN were determined by its green fluorescence after 10 min incubation at 37°C (far right dot plot) and. **(B)** PBMC were analyzed for expression pattern of specific surface marker using fluorochrome conjugated specific monoclonal antibodies (CD4 and CD8 presented as example) allowing the identification and quantification of lymphocyte subsets. **(C)** overlay histogram demonstrating the shift of fluorescence of cells with or without 12-O-tetradecanoylphorbol-13-acetate (TPA) due to oxidation of dihydrorhodamine by intracellular reactive oxygen species (ROS). **(D)** overlay histogram illustrating the shift of fluorescence of cells incubated with fluorescence-labelled bacteria on ice (control) or at 37°C to quantify the extent of phagocytosing capacity. Phagocytosis as well as the capacity to form intracellular ROS were determined by analyzing the percentage of fluorescent cells and by measuring their mean fluorescence intensity (MFI).

Phagocytic activity of PMN and peripheral blood mononuclear cells (PBMC) was investigated using the PHAGOTEST™ reagent kit (Glycotope Biotechnology, Heidelberg, Germany) according to the manufacturer’s protocol. This assay was previously tested for bovine blood samples and published as an applicable method to study phagocytosis in bovine leukocytes (34). In brief, heparinized whole blood was incubated with fluorescein (FITC) labeled opsonized *E. coli* bacteria solution for 10 min at 37°C followed by a quenching and washing procedure. A negative control sample was placed on ice for 10 min. After lysis of erythrocytes, all samples were stained with propidium iodide to discriminate leukocytes from bacteria (**Figure 2**). Cells were analyzed in duplicates using flow cytometry (FACSCanto™II, BD Biosciences, San Jose, CA, USA). For this, PMN and PBMC were gated according to their size and granularity based on measurements of forward and side scatter (**Figure 2A**). At least 10,000 cells per sample were evaluated. Results were expressed as percentage of phagocytosing PMN or PBMC and as calculated absolute phagocytosing PMN and PBMC by offsetting with cell counts. Additionally, the mean fluorescent intensity (MFI) was documented indicating cell phagocytic capacity.

#### 2.2.3 Reactive Oxygen Species Production

The capacity of PMN and PBMC to produce ROS was analyzed by flow cytometry based on the intracellular oxidation of the non-fluorescent dye dihydrorhodamine-(DHR)-123 to the fluorescent rhodamine-123 (R123) by hydrogen peroxide. In addition to an unstimulated sample quantifying basal ROS formation, a stimulation with the protein kinase C activation agent 12-O-tetradecanoylphorbol-13-acetate (TPA) was used to induce a respiratory burst by NADPH-oxidase activity. Briefly, EDTA whole blood was incubated with 40 µM DHR solution with or without 30 nM TPA supplementation for 15 min at 37°C. Erythrocytes were lysed for 10 min using lysis buffer (BD Pharm Lyse™, BD Bioscience, San Jose, CA, USA). After centrifugation (250 x *g*, 5 min, 4°C), cells were resuspended in with Hepes-buffered saline (HBS, 14 mM Hepes, 0.9% NaCl) and analyzed in duplicate by flow cytometry (FACSCanto™ II, BD Biosciences, San Jose, CA, USA). PMN and PBMC were gated according to their size and granularity based on measurements of forward and side scatter (**Figure 2A**). At least 10,000 cells were evaluated. The percentage of cells expressing rhodamine 123 fluorescence (R123^+^) was recorded. Additionally, the MFI of ROS-producing cells was used as indicator of cell individual capacity to produce ROS. TPA-induced oxidative burst capacity as well as basal ROS production in unstimulated PMN and PBMC were documented. Additionally, a stimulation index was calculated by dividing the TPA-stimulated by the non-stimulated fraction. Finally, percentages of R123^+^ cells were offset against the results of the automated cell counting and the absolute numbers of R123^+^ PMN or PBMC were calculated.

#### 2.2.4 Phenotyping of Lymphocyte Subsets

For immunophenotyping of lymphocyte subsets in EDTA whole blood specific mouse anti-bovine monoclonal antibodies (mAb, all purchased from AbD serotec, Bio-Rad laboratories GmbH, Feldkirchen, Germany) were used. To quantify T-helper (CD4^+^) and cytotoxic (CD8^+^) T-cells a dual staining procedure was used (CD4: FITC, CC8, IgG2a and CD8a: RPE, CC63, IgG2a). Expression of CD25^+^ was applied to identify T-regulatory cells (Treg) using CD4: FITC (CC8, IgG2a) and CD25: RPE (IL-A111, IgG1) in a dual staining. To define memory T-cells blood samples were triple stained with CD4: FITC (CC8, IgG2a), CD8: Alexa Fluor^®^ 647 (CC63, IgG2a), and CD45_RO :_ RPE (IL-A116, IgG3). B-lymphocytes (CD21^+^) and monocytes (CD14^+^) were identified using a dual staining with CD21: PE (CC51, IgG2b) and CD14: FITC (CC-G33, IgG1). Staining controls included cells stained with appropriate isotype control antibodies (Mouse IgG2a negative control: RPE clone OX-34, Mouse IgG2b negative control: FITC, A647, Mouse IgG1 negative control: FITC, Mouse IgG3 negative control: RPE) purchased from AbD serotec, Bio-Rad laboratories GmbH, Feldkirchen, Germany. Following antibody staining, erythrocytes were lysed using lysis buffer (BD FACS™ Lysing Solution, BD Biosciences, San Jose, USA) for 10 min on a shaker. After centrifugation (250 x *g*, 5 min, 4°C), cells were washed with HBS and measured using flow cytometry (FACSCanto™II, BD Biosciences, San Jose, CA, USA). To set up the flow cytometer for analysis, the population of PBMC was defined on the basis of their forward and side scatter properties while the target cell population was defined according to the gating strategy shown in **Figure 2**. Compensation of the spillover of all fluorochromes (FITC, PE, and Alexa Fluor^®^ 647) was performed with the BD FACS Diva software (BD Biosciences, San Jose, USA) and regular quality controls were performed using CS&T research beads (BD Biosciences, San Jose, USA). A total of at least 10,000 cells was counted. The percentage of different lymphocyte subsets are expressed as the proportion of PBMC or calculated as absolute numbers by offsetting with cell counts (lymphocytes + monocytes) determined by an automated cell analyzer (Celltac-α, MEK 6450, Nihon Kohden, Qinlab Diagnostik, Weichs, Germany). Additionally, the ratio between CD4^+^ and CD8^+^ T-cells was calculated.

#### 2.2.5 Peripheral Blood Mononuclear Cells Proliferation

Cell metabolic activity and Concanavalin A (ConA)-stimulated proliferation of PBMC were evaluated using the Alamar Blue (AB) assay which is based on the reduction of the non-fluorescent resazurin to the fluorescent molecule resorufin by metabolically active cells. For this, PBMC were isolated by density gradient centrifugation (Biocoll 1.077 g/ml, Biochrom AG, Berlin, Germany) from heparinized blood samples. The buffy coat was resuspended in Roswell Park Memorial Institute (RPMI)-medium enriched with 5% fetal bovine serum, 1 M HEPES buffer, 2 mM L-glutamine, 5 mM β-mercaptoethanol, 10,000 U/ml penicillin and 10 mg/ml streptomycin solution. All medium components were purchased from Biochrom AG, Berlin, Germany. Cell viability was determined by trypan blue exclusion technique and cell numbers were counted using a Neubauer counting chamber. Cells were incubated in a 96-well microplate (10^5^ cells/well) with or without ConA (2.5 µg/ml; Sigma-Aldrich, Steinheim, Germany) stimulation for 69.5 h at 37°C and 5% CO_2_, followed by incubation with Alamar Blue (AbD Serotec, Oxford, UK) in a ratio of 1:10 for 2.5 h. The fluorescence of resorufin was measured at 540 nm (excitation) and 590 nm (emission) using a photometer (Tecan infinite M200, Tecan, Grödig, Austria). Results of fivefold determination were calculated and expressed as stimulation index (SI).

#### 2.2.6 Cortisol

For cortisol analyses serum was gained by centrifugation of blood (15 min, 15°C, 1950× g, Varifuge 3.0, Heraeus, Hanau, Germany) after incubation for 30 min at room temperature and 30 min at 30°C and then stored at -80°C until measurement. The concentration of serum cortisol was measured at selected sampling points (-42 d, -14 d, -7 d, 0.5 h, 2 h, 4 h, 6 h,12 h, 24 h, 48 h, 7 d, 14 d, 21 d, 28 d, 42 d, 56 d, 100 d and 110 d) by radioimmunoassay according to the manufacturer’s protocol (Cortisol RIA Kit, Beckman Coulter, Krefeld, Germany). In brief, 50 µl of the sample or the control were incubated for 1 h at room temperature with 500 µl of ^125^I-labeld cortisol tracer or for the total count only with 500 µl tracer. Afterwards, bound and total cortisol were determined, and concentrations calculated with a standard curve.

### 2.3 Statistical Analysis

Statistical analysis was performed using the PROC MIXED procedure of SAS software package (Version 9.4, SAS Institute Inc., Cary, NC, USA) with the restricted maximum likelihood method. The model included group CON or CAR, time (experimental d relative to parturition) and the interaction between group and time as fixed effects. The values from d -42 were considered as co-variates. The choice of covariance structure (compound symmetry, autoregressive or unstructured) for the respective parameter were based on the smallest Akaike information criterion for a finite sample size (AICC). Statistical differences were declared significant at p ≤ 0.05 and from 0.05>0.10 as tendency. Additionally, differences of means were considered to be significant at p-values ≤ 0.05 using Tukey’s t-test. All results were presented as least square (LS)-means and pooled standard errors (PSE) were stated.

Correlations were calculated with Spearman’s rank correlation using JMP^®^ Pro (version 15, SAS Institute GmbH, Heidelberg, Germany) for the period from day 42 *ap* to 72 h *pp*.

## 3 Results

### 3.1 Hematology

**Figure 3:**
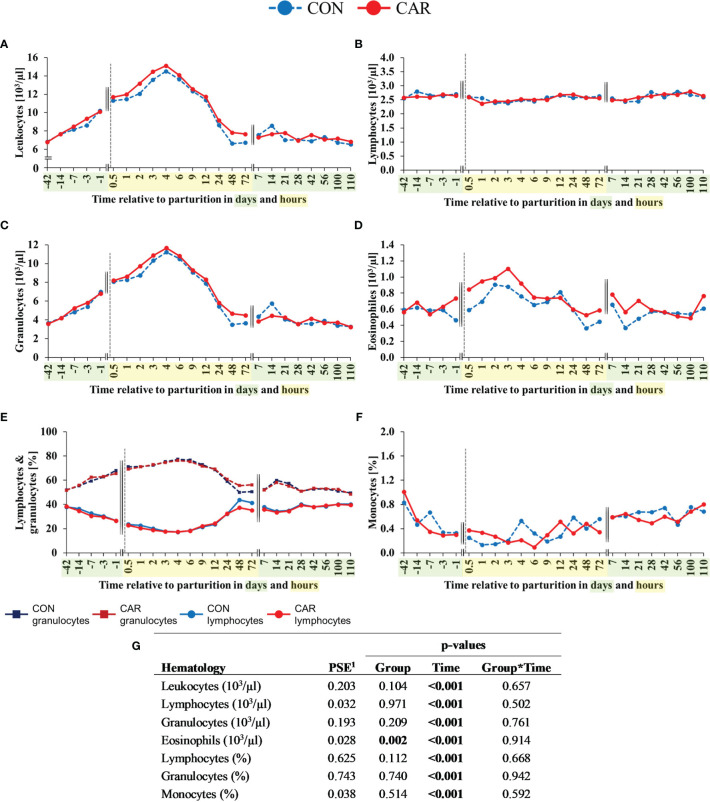
White blood cell counts of dairy cows fed a diet supplemented with (CAR) or without (CON) L-carnitine from 6 weeks before until 15 weeks after calving. **(A)** total leukocyte count, **(B)** lymphocyte count, **(C)** granulocyte count, **(D)** eosinophil count, **(E)** percentage of lymphocyte and granulocyte and **(F)** percentage of monocytes are determined by an automated cell analyzer. **(G)** Data statistics. Data are shown as least square means. ^1^pooled standard error.

**Figure 4:**
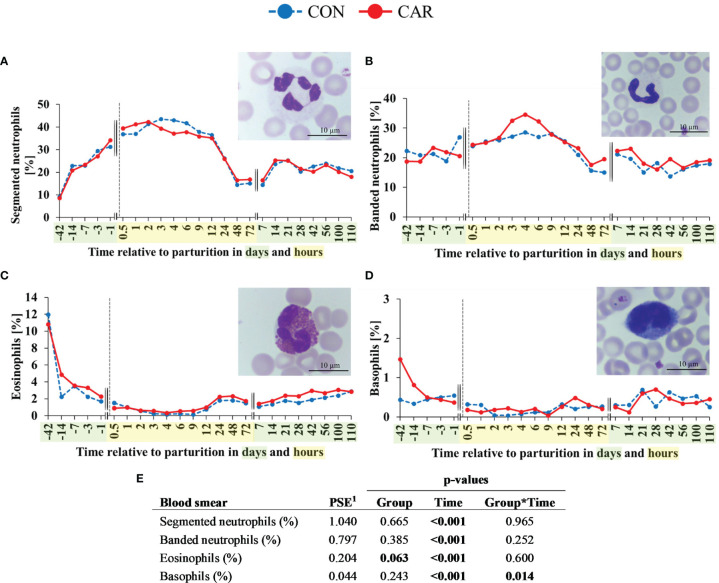
Quantification and different maturation stages of peripheral granulocytes of dairy cows fed a diet supplemented with (CAR) or without (CON) L-carnitine from 6 weeks before until 15 weeks after calving. **(A)** segmented neutrophils, **(B)** banded neutrophils, **(C)** eosinophils and **(D)** basophils counted manually on stained blood smears. **(E)** Data statistics. Data are shown as least square means; representative microscopic images of different granulocyte subsets are included respectively. ^1^pooled standard error.

The most pronounced changes in white blood cell count occurred before and within the first 4 h after calving and, with exception of the eosinophil and basophil granulocyte count and percentage, they were unaffected by L-carnitine supplementation. Comparative results of WBC differentiation were obtained by using an automated analyzer (**Figure 3**) and additionally by manual differentiation of blood smears (**Figure 4**).

For both groups equally, total leukocyte counts (**Figure 3A**) showed a constant increase to more than double concentration from d 42 *ap* until 4 h *pp* where it reached its maximum. Subsequently, it returned to initial level at 48 h *pp* and remained there until the end of the trial. The lymphocyte count (**Figure 3B**) showed significant, but only minor variations over the entire trial period, with the minimum at 3 h *pp* (2.40 x 10^3^/µl) and the maximum count on d 100 *pp* (2.76 x 10^3^/µl). Granulocyte count (**Figure 3C**) reflected the course of the total leukocytes and increased by three times from d 42 *ap* until its maximum at 4 h *pp.* Afterwards, the cell numbers decreased to the initial level till 48h remained until the end of the trial on d 110 *pp*. Percentage of lymphocytes (**Figure 3E**) decreased continuously from d 42 *ap* (38.17%) to the lowest point at 4 h *pp* (18.34 x 10^3^/µl). Afterwards, the percentage increased up to the initial level 72 h *pp* (38.33 x 10^3^/µl) and remained unchanged until the end of the trial. The percentage of granulocytes (**Figure 3E**) showed an opposite trend, as it increased from 42 *ap* (51.92%) and peaked at 4 h *pp* (76.75%), followed by a decrease until 48 h *pp* to the initial level (52.78%) until the end of the trial. In both feeding groups, numbers of eosinophils increased from d 1 *ap* to 2 h *pp* (CON by 2.75% points, CAR by 0.72% points) and reached their maximum there (**Figure 3D**). The mean eosinophilic granulocyte count of CAR was significantly higher (14%) over time as compared to that of CON (p=0.002) and also the mean percentage of eosinophils of CAR was 11% higher (p=0.049) than that of CON (**Supplementary Table 1**). While the monocyte count (**Supplementary Table 1**) remained unaffected by calving or supplementation, the percentage of monocytes (**Figure 3F**) decreased continuously by 80% from the beginning at d 42 before until 3 h after calving and started to increase nearly fourfold until the end of the trial irrespective of the supplementation.

Results of manual differentiation of WBC using blood smears were largely consistent with the automated quantification of blood cell populations. The determination of the maturation stage of neutrophilic granulocytes indicated a constant increase in mature granulocytes, as identified by a segmented nuclear structure, from d 42 *ap* to 2 h *pp* and a strong decrease after 12 h *pp* (**Figure 4A**) unaffected by CAR supplementation. The cell count dropped till 48 h *pp* and increased again until d 14 *pp* to a level that remained unchanged until the end of the trial. The proportion of immature neutrophilic granulocytes identified by a banded nuclear structure (**Figure 4B**) remained on the initial level until 2 h *pp* and increased until 4 h *pp.* This increase appeared more pronouncedly in the CAR supplemented cows, but without statistically significant differences between the groups. After the baseline level was reached 48 h *pp*, it remained there until the end of the trial. The proportion of eosinophilic granulocytes (**Figure 4C**) decreased significantly from d 42 *ap* (CAR 11%, CON 12%) until 4 h *pp* (CAR 0.3%, CON 0.2%) and recovered slightly until 24 h *pp* to a level that remained unchanged until the end of the trial. As already shown in the automated cell counts, the CAR group tended to have a higher (about 20%) proportion of eosinophils than the CON group (p=0.063). The proportion of the basophilic granulocytes (**Figure 4D**) in CAR cows (1.47%) was three times higher on d 42 *ap* compared to CON cows (0.44%), despite the inclusion of d 42 *ap* as a covariate. The basophils in CAR cows decreased significantly until 7 d *ap* whereas the proportion of CON cows stayed almost unchanged on the initial level until the end of the trial. The significant interaction between group and time was mainly determined by the initially high individual variation.

The curve progression of the lymphocyte and monocyte proportions (**Supplementary Table 1**) fluctuated over time and was in good accordance with the results of the cell count measurement using an automated cell analyzer (**Figures 3E, F**).

### 3.2 Functional Properties of Leukocytes

All parameters of leukocytes´ phagocytosis activity and intracellular ROS production were changed over time (p<0.001).

**Figure 5:**
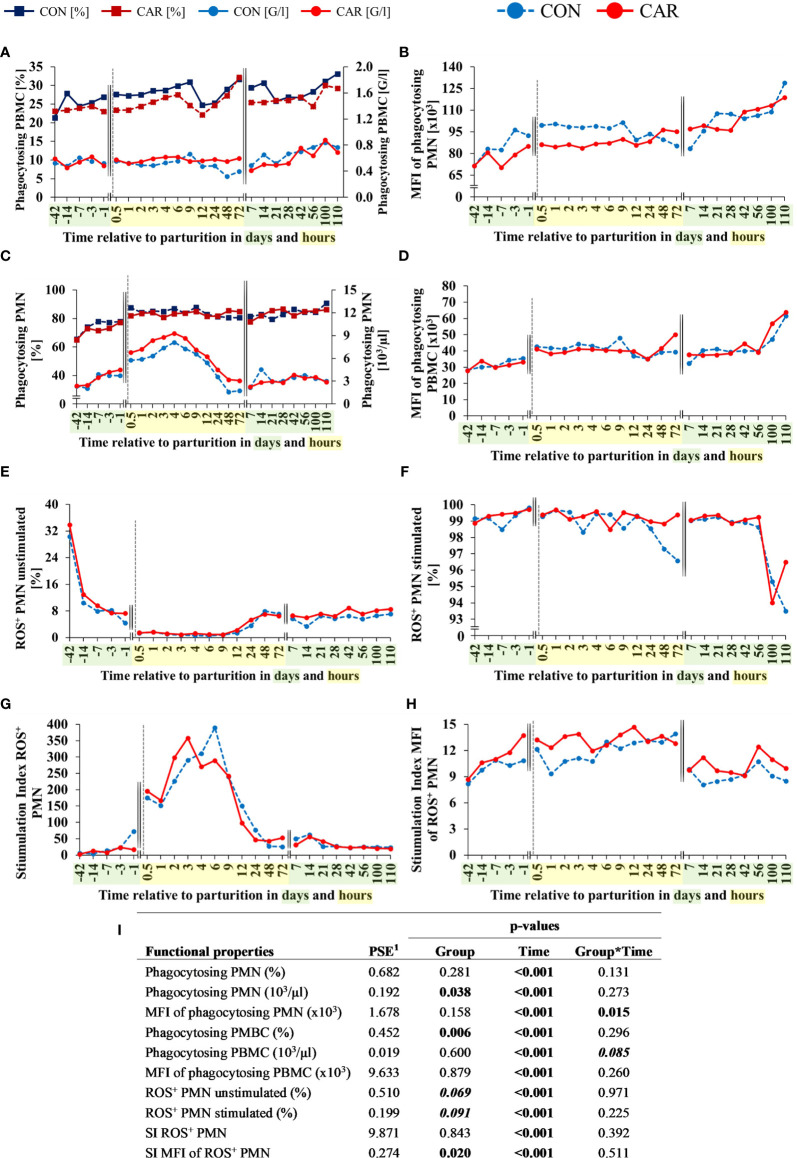
Functional properties of leukocytes of dairy cows fed a diet supplemented with (CAR) or without (CON) L-carnitine from 6 weeks before until 15 weeks after calving. **(A)** percentages and calculated numbers of phagocytosing polymorphonuclear leukocytes (PMN), **(B)** mean fluorescence intensities (MFI) of phagocytosing PMN, **(C)** percentages and calculated numbers of phagocytosing peripheral blood mononuclear cells (PBMC), **(D)** MFI of phagocytosing PBMC, **(E)** percentages of unstimulated reactive oxygen species (ROS^+^) producing PMN and **(F)** percentages of 12-O-tetradecanoylphorbol-13-acetate (TPA)-stimulated ROS^+^ PMN were determined by flow cytometry. Calculated stimulation index (SI) of percentage **(G)** and MFI **(H)** of stimulated and unstimulated ROS^+^ PMN. **(I)** Data statistics. Data are shown as least square means or as calculated numbers by offsetting results against to those of the absolute cell count. ^1^pooled standard error.

About 65% of all PMN were able to phagocytose FITC-labelled bacteria at d 42 *ap*. The percentage of phagocytosing PMN (**Figure 5A**) increased from the beginning of the trial until calving and 0.5 h *pp* by about 29% in both groups independent of CAR supplementation and remained unchanged until the end of the trial. Calculated absolute phagocytosing PMN also increased from the beginning until 4 h *pp* by 70% (CON) and 75% (CAR) (**Figure 5A**). A significant CAR effect (p=0.038) resulted from increased numbers of phagocytosing PMN 0.5 until 72 h *pp* in CAR supplemented cows compared to non-supplemented cows. In parallel to the proportion and number of phagocytosing cells, the phagocytic capacity, as determined by the MFI of all phagocytosing PMN (**Figure 5B**) also increased in both groups until calving, although the CAR supplemented cows remained at a significantly lower level within 24 h *pp*. Subsequently, the capacity of PMN increased continuously until the end of the trial, without any further CAR effect. An effect of CAR supplementation is expressed in the percentage of phagocytosing PBMC (**Figure 5C**) which was significantly lower in CAR than in CON cows, most pronounced from d 1 *ap* to 12 h *pp*. The capacity of phagocytosing PBMC (**Figure 5D**) increased in both groups slightly until 0.5 h *pp* and remained on this level until d 56 *pp*. Afterwards, it increased again significantly until the end of the trial. However, when calculating the percentages of phagocytosing PBMC with results of the automated cell counting (lymphocytes + monocytes), no significant CAR effect can be detected (**Figure 5C**).

The phagocytosis-independent production of intracellular ROS was determined by flow cytometry using a fluorescent dye-based assay. Time course of changes in unstimulated/basal ROS-formation in PMN is shown in **Figure 5E**, demonstrating a continuous and significant decrease by 95.8% from d 42 *ap* until calving and 0.5 h *pp* in both groups. This lower level persisted until 12 h *pp*, recovered slightly and remained almost unchanged until the end of the experimental trial. Compared to CON, proportion of ROS-forming PMN was affected by CAR supplementation (p=0.069), resulting in tendentially higher levels in CAR cows. The MFI of unstimulated PMN (**Supplementary Table 2**), indicating the level of ROS-production per cell, showed also a tendency between the groups (p=0.088), indicated by a higher value at 1 h *pp* in CON cows according to a significant trend by *post-hoc* test. To determine the efficiency of leukocytes to perform an oxidative burst, TPA was used in order to activate the responsible enzyme system to generate ROS. During the period from d 42 *ap* to d 56 *pp*, 99% of all PMN could be activated by TPA (**Figure 5F**). Smaller, but non-significant proportions of stimulated PMN were found from 24 h to 72 h after calving in the CON-group and a weak trend between the groups was detectable (p=0.091). The MFI of TPA-stimulated ROS^+^ PMN (**Supplementary Table 2**) was unaffected by CAR supplementation and fluctuated slightly over time (p<0.001). A calculated stimulation index (SI) of the percentage of ROS^+^ PMN (**Figure 5G**) demonstrated in both groups a significant increase within 12 h after calving, followed by a decrease to an initial level at 24 h *pp* which was then maintained until the end of the trial. The maximum of SI was between 3 h (CAR) and 6 h (CON), whereby CAR-supplementation had no significant effect. The calculated SI of MFI values of ROS^+^ PMN (**Figure 5H**) illustrated an increased capacity for CAR-supplemented cows around calving. This enhanced ability was demonstrated between d 3 *ap* and 4 h *pp*.

Time course of changes in variables of ROS production in PBMC is displayed in **Supplementary Table 2**. Proportion of basal and stimulated PBMC, as well as MFI of these cells, varied significantly over time, with pronounced changes around parturition and without any effect of CAR supplementation. Calculated stimulation indices are shown in **Supplementary Table 3** and varied also significantly over time without CAR influence.

Percentages of ROS^+^ PMN as well as ROS^+^ PBMC determined by flow cytometry were offset against the values of the automated cell counting (granulocytes or lymphocytes + monocytes, respectively) to obtain numbers of ROS-formatting cells. The courses and all results of statistical analyses are identical with those of the percentages and are shown in **Supplemental Figure 1**.

### 3.3 Phenotyping of Leukocyte Subsets

**Figure 6:**
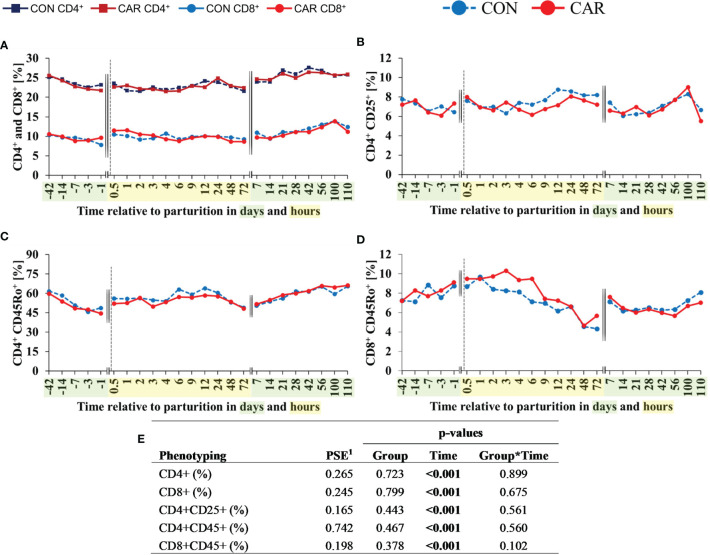
Flow cytometric quantification of leukocyte subpopulations of dairy cows fed a diet supplemented with (CAR) or without (CON) L-carnitine from 6 weeks before until 15 weeks after parturition. **(A)** T-helper (CD4^+^) and cytotoxic T-cells (CD8^+^), **(B)** regulatory T-cells (Treg, CD4^+^CD25^+^), **(C)** memory T-helper cells (CD4^+^CD45Ro^+^), and **(D)** memory cytotoxic T-cells (CD8^+^CD45Ro^+^) were expressed as percentages of total population of peripheral blood mononuclear cells (PBMC) identified by its relative size and granularity. **(E)** Data statistics. Data are shown as least square means. ^1^pooled standard error.

All phenotypes of the leukocytes changed over time (p<0.001) but were unaffected by treatments or their interaction (**Figure 6**).

The percentage of T-helper (CD4^+^) and cytotoxic T (CD8^+^) lymphocytes is expressed as the proportion of all PBMC characterized by their flow cytometric forward and side scatter properties. While the percentage of T-helper cells (**Figure 6A**) decreased from d 42 *ap* until 4 h *pp* and then stayed at the initial level until the end of the trial, the percentage of cytotoxic T-cells (**Figure 6A**) as well as the ratio of CD4^+^ to CD8^+^ (**Supplementary Table 4**) fluctuated in an undirect manner over time. Using additional cell surface markers, further subsets of T-lymphocytes were defined and expressed as the proportion of CD4^+^ or CD8^+^ cells, respectively. The percentage of Treg cells (**Figure 6B**) defined by a combination of CD4^+^ and CD25^+^ expression showed minor shifts over the complete experimental time in the range of 6.1 to 8.6%. The percentage of memory T-helper cells, indicated by the expression of CD4^+^ and CD45Ro^+^ (**Figure 6C**) decreased continuously from d 42 *ap* to 1 d *ap* equally in both groups, recovered *pp* and stayed at baseline level. The percentage of memory cytotoxic T-cells (**Figure 6D**) defined by the expression of CD8^+^ and CD45Ro^+^ remained largely at baseline level from d 42 *ap* to 1 h *pp*, then decreased by half until 48 h *pp* and returned to the initial level at d 7 *pp*. For CD4^+^ and CD8^+^ phenotypes, the MFI, as parameter of the expression density of surface marker, showed significant but only minor shifts over the whole trial period, as displayed in **Supplementary Table 4**.

**Figure 7:**
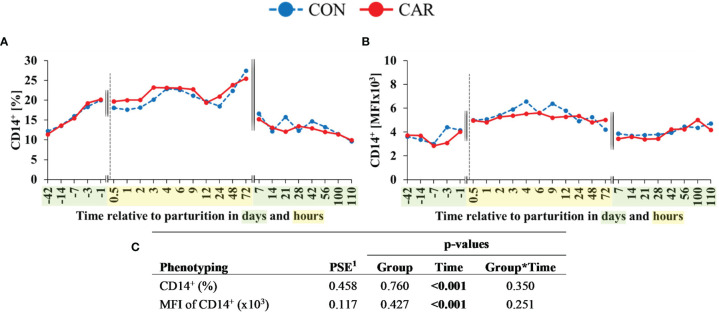
Flow cytometric quantification of monocyte population in dairy cows fed a diet supplemented with (CAR) or without (CON) L-carnitine from 6 weeks before until 15 weeks after parturition. **(A)** percentage of cells expressing monocytes (CD14^+^) and **(B)** mean fluorescence intensity (MFI) of monocytes (CD14^+^). **(C)** Data statistics. Data are shown as least square means. ^1^pooled standard error.

The percentage of CD14^+^ cells (**Figure 7A**), corresponding to monocytes, doubled from 42 d *ap* to 1 d *ap* and increased to the maximum at 72 h *pp*. At d 7 *pp*, this cell population exhibited the initial level once more and remained statistically unchanged until the end of the trial. The expression of CD14^+^ (**Figure 7B**) increased by 47% from one d *ap* to 4 h *pp*, persisted on this higher level until 72 h *pp* and decreased again at d 7 *pp* to the initial level until the end of the trial. The percentage of B-cells (CD21^+^) and their MFI (**Supplementary Table 2**) fluctuated only a little over the whole trial period.

The percentages of all lymphocyte subsets were additionally offset against the absolute cell counts (lymphocytes + monocytes) from the hematological analysis and are shown in **Supplemental Figure 2**. The results are largely consistent with those of the proportions in curve progressions, and the results of the statistical analyses are found to be identical with the exception of CD14^+^ cells.

### 3.4 Peripheral Blood Mononuclear Cells Proliferation

**Figure 8:**
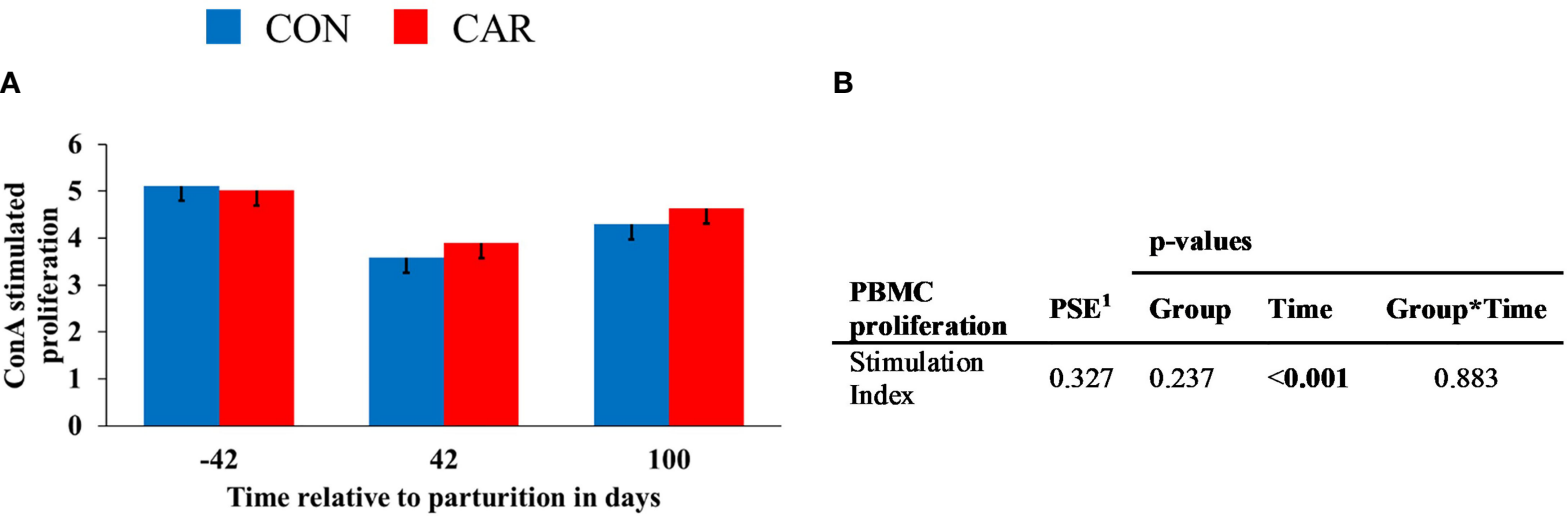
ConA stimulated proliferation of peripheral blood mononuclear cells (PBMC) of dairy cows fed a diet supplemented with (CAR) or without (CON) L-carnitine from 6 weeks before until 15 weeks after parturition. **(A)** Stimulation Index (SI) is defined as the ratio between the fluorescence in Alamar Blue assay of concanavalin A-stimulated PBMC and non-stimulated PBMC. **(B)** Data statistics. Data are shown as least square means. ^1^pooled standard error.

The ConA stimulated lymphocyte proliferation changed over time (p<0.001) and showed no influence of treatment on the SI. SI (**Figure 8**) decreased by 26% from d 42 *ap* to 42 *pp* and then increased again by 19% to d 100 *pp*.

### 3.5 Cortisol

**Figure 9:**
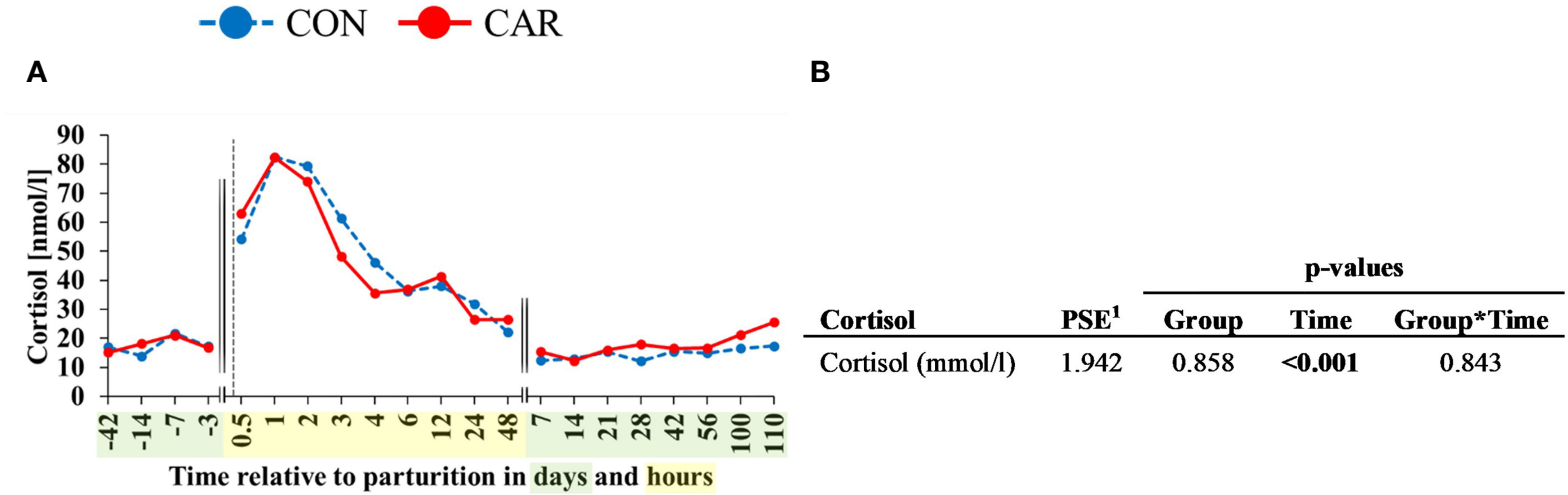
**(A)** Effects of dietary L-carnitine on serum cortisol of dairy cows fed diet supplemented with (CAR) or without (CON) L-carnitine from 6 weeks before until 15 weeks after calving. **(B)** Data statistics. Data are shown as least square means. ^1^pooled standard error.

The serum cortisol concentration was affected by calving (p<0.001) but was not influenced by treatment or the interaction of time and treatment. The *ap* cortisol concentration (**Figure 9**) remained on the initial level (16.12 nmol/l) until d 3 *ap*. Directly after calving (0.5 h *pp*) cortisol was increased significantly and reached its peak with 82.38 nmol/l at 1 h *pp*. Afterwards, it decreased until d 7 *pp* and remained on the initial level until the end of the trial.

## 4 Discussion

The transition period and early lactation are among the most intensive and challenging time a dairy cow faces in life. The ability to cope with the onset of milk production and the physiological stress of parturition by means of sufficient energy supply and fast recovery are critical for health stability and consistent performance in dairy cows. Supplementation of L-carnitine might improve the cellular energy situation during the stressful time of parturition due to its fundamental role in ß-oxidation of long chain fatty acids for energy production. Thus, the aim of the present study was to investigate the hypothesis that L-carnitine supplementation affected the leukocyte population of the cow around calving with consequences on the function of PMN and lymphocytes.

In result of this study, characteristic dynamics of white blood cell populations during the transition period were described. Overall, a continuous increase in the total leukocyte count from the beginning until 4 h *pp* and a drop to the initial level within 48 h *pp* was shown. In agreement with other studies (17, 35), this leukocytosis occurs as physiological response in preparation of and during parturition. While lymphocytes changed only slightly over time, the granulocyte population, consisting mainly of neutrophils, experienced marked neutrophilia throughout late pregnancy and parturition which had previously been described as associated with physiological stress and is constituting a typical finding for this period (24–26). Burton et al. (31) depicted the down-regulation of L-selectin expression on bovine neutrophils caused by increased cortisol level at parturition which might inhibit the immigration of neutrophilic granulocytes into the tissue. This suggestion is also supported by others who described a neutrophilia around parturition in dairy cows and assumed that this increase was caused by glucocorticoids (26) and resulted in a reduced migration of neutrophils immediately after calving (36). Earlier, some authors assumed that increased corticosteroid levels *pp* ensured that the production of neutrophil granulocytes in the bone marrow was increased and that demargination from the blood vessel wall occurred or a combination of both (37, 38). In order to investigate these assumptions, the determination of cell numbers using automated cell counters is not sufficient, since these devices cannot determine the maturation stages of the cells. Therefore, manual differentiation and counting of WBC were performed using stained blood smears. In the present trial the time course of segmented neutrophils which correspond to the matured neutrophils mimicked that of total granulocytes determined with the automated counter and, like these, increased continuously during late pregnancy. Only in response to parturition, the number of banded granulocytes which correspond to immature neutrophilic granulocytes was temporarily increased within 9 h *pp*, indicating a slightly increased release of neutrophilic granulocytes from the bone marrow. This could confirm that demargination of functional, matured neutrophils from the endothelium into the peripheral circulation might be most significant in early post-natal phase. In addition to this, the significantly increased secretion of cortisol right after parturition strongly reflected a state of physiological stress (39, 40) and supports the aforementioned connection between glucocorticoids and their influence on the neutrophilic granulocytes. A glucocorticoid induced down-regulation of surface adhesion molecule expression (31, 41) may thus also be involved in the observed neutrophilia. This study failed to demonstrate an effect of L-carnitine on either amount, differentiation or progression of neutrophilic granulocytes or the blood cortisol level.

The proportion of all populations of WBC are influenced in relation to parturition, but only for eosinophil and basophil granulocytes an L-carnitine effect could be detected. The results of automated cell counting showed a slight but significant increase of peripheral eosinophils directly before calving and in the early postnatal period in cows supplemented with dietary L-carnitine. In recent years, additional functions of eosinophils have been described (42), indicating that these cells not only play a primary role in host defense against pathogens, but also influence tissue remodeling and maintenance of homeostasis (43). Since, according to the current study, the most pronounced changes in this cell population occurred shortly before and after calving, it can be assumed that this might be mainly related to tissue repair and remodeling processes in direct response to parturition. It was precisely during this period that L-carnitine supplementation led to an additional increase in circulating eosinophils and, at the same time, to an increased number of platelets, which had previously been published as one result from this project (12). On the one hand, this finding confirms the described evidence for tissue healing processes after calving, which is in accordance with the reciprocal influence between eosinophils and platelets described in some studies, including the production of signaling molecules for the coagulation system mediated by eosinophils (44–46). On the other hand, an influence by L-carnitine at this level may indicate an enhanced support for tissue repair and recovery. A significant interaction (Group*Time) of the basophil proportions determined by manual differentiation can, however, not be plausibly explained and also showed no directional L-carnitine effect in this study. It can be assumed that this result was rather caused by the high individual variation at the beginning of the trial.

Previous studies on L-carnitine supplementation in dairy cows mainly addressed the influence of L-carnitine on performance-related parameters, whereas data on its effect on absolute numbers of leukocytes are still limited. There are only few studies in mammals that investigated effects of L-carnitine supplementation linked with blood cell quantities. For example, Strasser et al. (47) examined the effects of a combined administration of L-carnitine (150 mg/kg BW dissolved in drinking water) and dehydroepiandrosterone sulfate (DHEAS) on blood cells of aged Sprague–Dawley rats. They could not find any impacts of L-carnitine supplementation on WBC, lymphocytes and granulocytes, but they detected increased platelet counts, corresponding to lower mean platelet volume and discussed a decreased activation state of the cells. L-carnitine-mediated inhibition of platelet-activating factor synthesis in human neutrophils and platelets was described elsewhere (7–9, 48) and may confirm this assumption. In addition, Strasser et al. (47) reported decreased numbers of monocytes in their L-carnitine supplemented group, but contrary to this, no effect of L-carnitine on monocytes was found according to the present study.

Along with varying proportions of cell populations, the functional properties of blood cells are also affected in relation to calving. In this study, phagocytosis was determined *ex vivo* by ingestion of fluorescent bacteria into the cells and also the phagocytosis independent formation of ROS was analyzed using a whole blood assay. It was shown, that the proportion of phagocytosing PMN and simultaneously the capacity of these cells increased consistently during late pregnancy until calving, whereas in L-carnitine supplemented cows a higher number of phagocytosing PMN and a lower cellular uptake of bacteria were recorded around parturition. Accordingly, the current study in fact described a reduced capacity of PMN to phagocytose bacteria one week before to 9 h after calving in L-carnitine supplemented compared to non-supplemented cows. Almost simultaneously, these cells showed a significantly increased cellular efficiency to produce ROS after stimulation, as determined by an SI calculated from the unstimulated and stimulated fluorescence intensity of ROS-producing PMN. Although the unstimulated cellular ROS formation in PMN was significantly affected by calving in both groups and resulted in a strong decrease, L-carnitine supplementation tended to increase the proportion of ROS-producing PMN throughout the trial, indicating altered cellular activity in this group. It might be possible that the higher SI demonstrates a temporarily improved efficiency of PMN around parturition due to L-carnitine supplementation. It has to be remembered that in periods of physiological stress, like parturition, cellular activities change and inevitably also the activities of trans-membrane proteins. In general, a consideration of physiological circumstances of the animals and possible effects of L-carnitine supplementation seems to be important since many studies reported favorable impacts of L-carnitine supplementation in subjects with degenerative diseases or impaired membrane functions. Interesting, for example, is a study of Izgüt-Uysal et al. (49) which investigated L-carnitine effects on cells of aging rats. While aging processes lead to the impairment of various cellular functions, a supplementation of L-carnitine caused an improvement and a support of physiological functions of neutrophils.

Phagocytic NADPH-oxidase is a membrane bound enzyme complex responsible for oxidative burst activities and since it was shown that L-carnitine has membrane-stabilizing properties (50) and also prevents cellular membranes from damage due to oxidative stress (7, 8), it can be presumed that L-carnitine supports intracellular ROS generation and phagocytes function in a positive manner. The membrane-modifying effect of L-carnitine and its conjugated form, acylcarnitine, has been confirmed by several studies. In search of the mode of action, Butterfield and Rangachari (51) demonstrated that L-carnitine increased cytoskeletal protein-protein interactions on membranes of human erythrocytes leading to increased membrane stability.

In a number of tissue-related cells ROS-producing enzyme complexes are major regulators involved in cell differentiation and apoptosis due to cell signaling and regulation of gene expression (52, 53). Some *in vivo* and several *in vitro* studies demonstrated an antioxidative effect of L-carnitine supplementation resulting in reduced intracellular ROS production or modulation of NADPH oxidase activity (50, 54–56). The documented dynamics of ROS-forming bovine PMN around parturition in this study have been reported in comparable proportions by Bühler et al. (57) and showed very clearly the physiological effects of calving at the cellular level. Da Silva et al. (58) found also a decrease in ROS production of PMN from 7 d *ap* to 3 d *pp* and discussed the NEB as causative for this decrease. In the present study, a slight correlation between functionality of PMN and PBMC *ex vivo* and parameters of the energy metabolism (13) *in vivo* could be detected. A slightly positive correlation between NEB and ROS production of unstimulated PMN (r=0.228; p<0.001) indicates an influence at the energetic level.

Parameters on energy metabolism from the current project have already been published by Meyer et al. (13) and documented an influence of L-carnitine on the serum concentration of triglyceride (TG) as well as a tendency (p=0.06) of reduced NEFA levels, which is noticeable in a short period around calving. It is conceivable that a lower NEFA level at this phase in L-carnitine supplemented cows was due to increased transport of NEFA into the cells and subsequently into the mitochondrial matrix, as previously described in a study by Erfle et al. (59). They reported intravenous L-carnitine infusion lowering plasma concentrations of free fatty acids primary in the context of high initial concentrations due to ketotic conditions and suggested an enhanced transport to the mitochondria and subsequent oxidation of the fatty acids. In the present study, the NEFA level of non-supplemented cows tended to be higher especially at one d before up to 1 h after calving. To improve the energy supply during the transition phase, dairy cows mobilize adipose tissue providing TG and NEFA as major sources of energy for tissues and cells. Plasma NEFA potentially interact directly with blood cells, as described by Lacetera et al. (60) resulting in changes of leukocyte functions *inter alia* by modifying protein structure and function due to palmitoylation. Furthermore, an *in vitro* study (61) showed a concentration-dependent effect of NEFA on PMN viability and ROS-production. In fact, it is discussed that the reduced dry matter intake and the increased NEFA concentration lead to a temporary immunosuppression in dairy cows (21, 62). Correlation analysis of this study suggested a slightly positive association between NEFA and phagocytosing PMN (r=0.366; p<0.001) and also for the phagocytosing capacity (r=0.201; p<0.001) and ROS stimulation index (r=0.235; p<0.001) of PMBC. Therefore, it is conceivable that blood leukocytes in L-carnitine supplemented cows may have an improved energetic situation around calving, besides the fact that cells of non-supplemented cows were more stressed by the presence of more plasma NEFA.

The number and proportion of lymphocytes showed only minor shifts with a minimum at 3 h *pp*. This decrease directly *pp* was detected also in previous studies for cows and goats (24, 25) where these changes were discussed in the context of increased plasma corticosteroid levels *pp* (24) which was also observed in the present study. To provide a more detailed characterization of lymphocytes antibody staining was used to quantify the percentage of T-helper (CD4^+^), cytotoxic T-cells (CD8^+^), memory- (CD45^+^) and regulatory- (CD25^+^) T-cells as well as B-cells (CD21^+^) and monocytes (CD14^+^). All of these specific subpopulations showed significant fluctuations over time, without any effect of dietary L-carnitine. For T-helper cells and cytotoxic T-cells, the observed decline until parturition was often described for dairy cattle (62, 63). Such changes in PBMC subpopulations during the immediate periparturient period are discussed as assumed immune suppression in cows (26, 60, 64), but the detailed background is not known yet (62). In the present study, the results of phagocytic activity of PBMC documented an increase around parturition, whereas the percentage of basal ROS producing PBMC decreased from 3 d *ap* until 48 h *pp*. At the same time, the MFI of these ROS producing PBMC changed only slightly during this period. This implies that fewer ROS producing PBMC were activated during the first hours *pp*, but arguing against an immune suppression at this time is the finding that the ability to produce ROS was maintained over time. Overall, it can be concluded that although all investigated cellular parameters were affected by parturition, no inhibitory effect on the proportion of phagocytosing cells, their ability to phagocytose bacteria, or on the ability of NADPH oxidase to induce an oxidative burst can be deduced in the current study.

The results from the PBMC proliferation assay demonstrated a reduced proliferation on 42 d *pp* compared to 42 d *ap*, indicating a different sensibility of PBMC. No effects of L-carnitine supplementation were detected, whereby earlier experiments had confirmed the ability of L-carnitine and acetyl-L-carnitine to enhance mitogen- and antigen-driven lymphocyte proliferation under *in vitro* conditions (64). In human immunodeficiency virus (HIV) - positive patients it was shown that lymphocyte proliferation was markedly increased in L-carnitine- or acetyl-L-carnitine preloaded lymphocytes. L-carnitine also protected age-related alterations in peripheral blood lymphocytes and improved their defective proliferative capability when such cells were exposed to oxidative stress (65). The lack of L-carnitine effects can possibly be explained by the fact that the studies described so far examined cells from immunosuppressed subjects already impaired in their function, whereas the present study used PBMC from physiological healthy cows.

The percentage of monocytes, measured with antibody staining, decreased *ap* and increased *pp.* Similar results for percentage of monocytes could be shown in Eger et al. (66) and Røntved et al. (67) whereby the results of the automated cell analyzer of our study showed different trends over time. A reason for this discrepancy seems to be the different reference values. While automated counting used the total WBC count as reference value, the flow cytometric measurement used the total PBMC. The percentage and expression of B-cells fluctuated only a little over the whole trial period. For B-cells around parturition the same was reported by Ohtsuka et al. (68) who compared two different nutritive conditions and the effect on PBMC in periparturient dairy cows (68). They started their observations in week 16 *ap* and ended in week 14 *pp* and took samples every 4 weeks. The number of B-cells also fluctuated only slightly over the whole trial period (68). Our findings and these of Ohtsuka et al. (68) were in contrast to those of Meglia et al. (26) who did observe changes in the number of B-cells in early lactation in dairy cows fed different nutritional levels during the dry period. According to Meglia et al. (26) the proportions of B-cells increased in early and late lactation and were discussed in relation to changes in hormonal status. It is often claimed that the hormonal changes around parturition are responsible for changes in cell counts which has also been mentioned earlier in this discussion. Glucocorticoids for example induce leukocytosis, neutrophilia, monocytosis and lymphopenia (69) which are observed around parturition. In general, several hormones of maternal, placental and/or fetal origin seem to play key roles in the regulation of different lymphocyte subpopulations (70). Increased cortisol and placental corticotropin-releasing hormones are essential for the parturition initiation in addition to functional progesterone withdrawal and estrogen activation (70). The complex endocrine regulatory network also includes oxytocin, prostaglandins, nitric oxide, cytokines and immune cells (70) whereby the exact mechanism remains unknown. In the present study, a positive correlation between the stress hormone cortisol could be shown for the proportion of phagocytosing PMN (r=0.314; p<0.001), the phagocytic capacity of PBMC (r=0.210; p<0.001) and also for WBC (r=0.340; p<0.001) and granulocytes (r=0.382, p<0.001). A slightly negative correlation was shown for the number of lymphocytes (r=-0.429; p<0.001).

In summary, the presented results demonstrated that a supplementation of 25 g rumen-protected L-carnitine per cow and d during the challenging phase of late gestation and parturition affects different blood cell populations, as seen mainly in functional parameters of leukocytes. There is evidence that L-carnitine can be effective during this time when blood cells are affected at an energetic level or by hormonal influences. Besides an increased number of peripheral eosinophils in L-carnitine-supplemented cows, which may positively influence tissue healing, these cows showed an increased efficiency of intracellular ROS formation in stimulated PMN, and higher numbers of phagocytosing PMN. At the same time, decreased phagocytic activity in leukocytes was shown which could be due to an affected cell activity. In addition, L-carnitine-supplemented cows tended to have lower plasma NEFA levels than non-supplemented cows immediately before calving which may indicate enhanced translocation of NEFA into mitochondria by L-carnitine. In this context, an altered intracellular L-carnitine level could also be important for changed cellular functions, but this was not investigated here. Finally, according to this study, a short period around calving (immediately before to 4 h after calving) proved to be a sensitive period in which L-carnitine administration was effective and should be investigated more intensively in further studies.

## Data Availability Statement

The original contributions presented in the study are included in the article/**Supplementary Material**. Further inquiries can be directed to the corresponding author.

## Ethics Statement

The animal study was reviewed and approved by Lower Saxony State Office for Consumer Protection and Food Safety, AZ33.19-42502-04-16/2378.

## Author Contributions

Conceptualization: SD, JF, and KH. Formal analysis: SUK. Investigation: SUK, JM, KH, JF, JK, SK, and AW. Resources: SUK. Data curation: SUK. Writing original draft preparation: SUK. Review and editing: SUK, JM, JF, SB, JK, SK, AW, JR, UM, KH, and SD. Visualization: SUK and JM. Supervision: JF, SK, SD, and KH. Project administration: KH and SD. Funding acquisition: KH and SD. All authors contributed to the article and approved the submitted version.

## Funding

This experiment is part of the cooperation project MitoCow funded by the German Research Foundation (DFG, 202989534). SUK was supported by a scholarship of the H. Wilhelm Schaumann Foundation.

## Conflict of Interest

The authors declare that the research was conducted in the absence of any commercial or financial relationships that could be construed as a potential conflict of interest.

## Publisher’s Note

All claims expressed in this article are solely those of the authors and do not necessarily represent those of their affiliated organizations, or those of the publisher, the editors and the reviewers. Any product that may be evaluated in this article, or claim that may be made by its manufacturer, is not guaranteed or endorsed by the publisher.
