# Supplementary material for: Dietary L-Carnitine Affects Leukocyte Count and Function in Dairy Cows Around Parturition
Source: Front Immunol. 2022 Mar 16;13:784046. doi: 10.3389/fimmu.2022.784046 (PMC8965741; doi:10.3389/fimmu.2022.784046)
Supplement: Supplementary file 1 [file DataSheet_1.docx]

**Supplementary Material**

**Supplementary Table 1.** Effects of dietary L-carnitine on monocytes and eosinophils, measured with automated cell analyser, on dairy cows fed a diet supplemented with (CAR) or without (CON) L-carnitine from 6 weeks before until 15 weeks after calving.

| **Days/ Hours^1^** | **Monocytes* (10^3^/µl)** | **Eosinophil* (%)** | | **Lymphocytes^#^ (%)** | **Monocytes^#^ (%)** |
| --- | --- | --- | --- | --- | --- |
|  |  | **CON** | **CAR** |  |  |
| -42 | 0.06 | 8.03 | 8.73 | 57.0 | 0.79 |
| -14 | 0.04 | 8.59 | 8.02 | 53.4 | 0.53 |
| -7 | 0.03 | 7.36 | 6.68 | 49.6 | 0.58 |
| -3 | 0.02 | 6.65 | 7.08 | 48.4 | 0.62 |
| -1 | 0.03 | 4.6,3 | 7.58 | 39.7 | 0.83 |
| 0.5 | 0.03 | 5.16 | 7.86 | 36.2 | 0.57 |
| 1 | 0.01 | 6.22 | 7.98 | 34.2 | 0.66 |
| 2 | 0.02 | 7.38 | 8.27 | 30.8 | 0.37 |
| 3 | 0.02 | 6.87 | 7.51 | 27.5 | 0.61 |
| 4 | 0.04 | 5.30 | 6.02 | 27.4 | 0.59 |
| 6 | 0.02 | 4.75 | 6.16 | 29.4 | 0.51 |
| 9 | 0.02 | 5.68 | 5.90 | 33.9 | 0.70 |
| 12 | 0.04 | 7.52 | 6.00 | 37.1 | 0.60 |
| 24 | 0.03 | 8.19 | 6.34 | 48.6 | 0.78 |
| 48 | 0.03 | 5.07 | 6.59 | 64.9 | 1.02 |
| 72 | 0.02 | 7.56 | 8.16 | 63.3 | 1.70 |
| 7 | 0.03 | 9.44 | 11.65 | 60.8 | 1.02 |
| 14 | 0.37 | 5.14 | 7.62 | 52.5 | 0.60 |
| 21 | 0.03 | 7.17 | 9.45 | 56.3 | 0.30 |
| 28 | 0.03 | 8.25 | 9.43 | 59.6 | 0.39 |
| 42 | 0.04 | 8.46 | 8.00 | 58.9 | 0.37 |
| 56 | 0.03 | 8.02 | 7.43 | 57.5 | 0.68 |
| 100 | 0.04 | 7.90 | 6.88 | 56.9 | 1.33 |
| 110 | 0.05 | 9.53 | 11.00 | 58.0 | 1.50 |
| p-Value |  | | | | |
| Group | 0.902 | **0.049** | | 0.117 | 0.362 |
| Time | 0.157 | **<0.001** | | **<0.001** | **<0.001** |
| Group*Time | 0.151 | 0.945 | | 0.892 | 0.738 |
| PSE^2^ | 0.004 | 0.359 | | 1.004 | 0.075 |

Data are shown as least square means. ^1^ day/hours relative to parturition, ^2^ pooled standard error, * measured with automated cell analyser, ^#^ measured on blood smears.

**Supplementary Table 2:** Effects of dietary L-carnitine on functional properties of leukocytes on dairy cows fed a diet supplemented with (CAR) or without (CON) L-carnitine from 6 weeks before until 15 weeks after calving.

| **Days/ Hours^1^** | **MFI^3^ of ROS^+4^ PMN^5^ unstimulated (x10^3^)** | | **MFI^3^ of ROS^+4^ PMN^5^ stimulated (x10^3^)** | **ROS^+4^ PBMC^6^ unstimulated (%)** | **ROS^+4^ PBMC^6^ stimulated (%)** | **MFI^3^ of ROS^+4^ PBMC^6^ unstimulated (x10^3^)** | **MFI^3^ of ROS^+4^ PBMC^6^ stimulated (x10^3^)** |
| --- | --- | --- | --- | --- | --- | --- | --- |
|  | **CON** | **CAR** |  |  |  |  |  |
| -42 | 14.1 | 13.9 | 104.8 | 1.48 | 32.2 | 5.09 | 13.3 |
| -14 | 11.9 | 10.1 | 99.1 | 0.99 | 38.5 | 6.87 | 17.1 |
| -7 | 10.2 | 10.7 | 103.4 | 2.46 | 44.7 | 5.85 | 13.6 |
| -3 | 10.8 | 9.7 | 101.4 | 2.52 | 55.8 | 5.82 | 12.8 |
| -1 | 11.2 | 8.8 | 101.0 | 1.89 | 69.0 | 6.22 | 11.5 |
| 0.5 | 10.2 | 11.1 | 106.1 | 0.94 | 63.8 | 5.85 | 12.0 |
| 1 | 15.6 | 12.3 | 106.0 | 1.48 | 66.3 | 7.03 | 12.4 |
| 2 | 13.5 | 9.2 | 104.8 | 0.98 | 65.9 | 7.08 | 11.5 |
| 3 | 13.6 | 9.5 | 106.4 | 0.63 | 70.7 | 6.00 | 12.9 |
| 4 | 12.9 | 12.8 | 105.8 | 1.17 | 71.5 | 5.95 | 12.4 |
| 6 | 11.4 | 11.2 | 109.2 | 0.65 | 69.7 | 7.16 | 13.1 |
| 9 | 11.3 | 8.7 | 105.6 | 0.70 | 61.9 | 8.68 | 11.7 |
| 12 | 9.8 | 9.0 | 109.8 | 1.13 | 60.5 | 6.76 | 11.6 |
| 24 | 9.4 | 8.89 | 104.3 | 1.03 | 39.4 | 6.03 | 14.5 |
| 48 | 9.8 | 9.0 | 109.8 | 0.55 | 29.5 | 6.04 | 17.3 |
| 72 | 9.6 | 10.3 | 113.5 | 1.09 | 33.0 | 5.45 | 15.5 |
| 7 | 12.9 | 13.6 | 104.2 | 2.26 | 42.0 | 5.83 | 15.9 |
| 14 | 15.4 | 11.9 | 102.5 | 1.26 | 38.5 | 6.62 | 12.6 |
| 21 | 12.8 | 11.3 | 97.7 | 1.04 | 38.4 | 6.44 | 9.5 |
| 28 | 12.1 | 10.9 | 93.8 | 0.13 | 29.4 | 8.27 | 12.6 |
| 42 | 11.5 | 12.3 | 96.3 | 0.49 | 26.7 | 7.46 | 11.0 |
| 56 | 10.2 | 8.7 | 98.7 | 0.15 | 30.2 | 7.00 | 9.4 |
| 100 | 10.6 | 8.9 | 89.2 | 0.12 | 26.9 | 7.37 | 10.6 |
| 110 | 11.0 | 10.3 | 91.6 | 0.27 | 22.5 | 5.69 | 11.1 |
| p-Value |  | | | | | | |
| Group | **0.088** | | 0.118 | 0.147 | 0.479 | 0.755 | 0.314 |
| Time | **<0.001** | | **<0.001** | **<0.001** | **<0.001** | **0.002** | **<0.001** |
| Group*Time | 0.585 | | 0.873 | 0.432 | 0.656 | 0.991 | 0.569 |
| PSE^2^ | 3.653 | | 10.383 | 0.193 | 1.604 | 2.549 | 4.903 |

Data are shown as least square means. ^1^ day/hours relative to parturition, ^2^ pooled standard error, **^3^**mean fluorescence intensity, ^4^ reactive oxygen species, ^5^ polymorphonuclear leukocytes, ^6^ peripheral blood mononuclear cell.

| 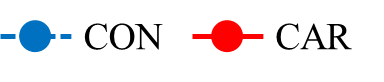 | |
| --- | --- |
| (A)  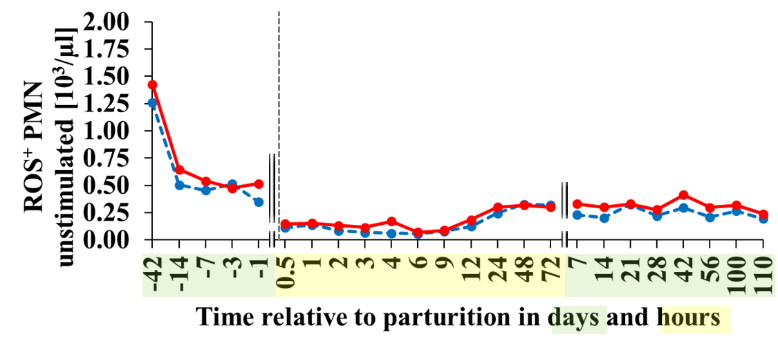 | (B)  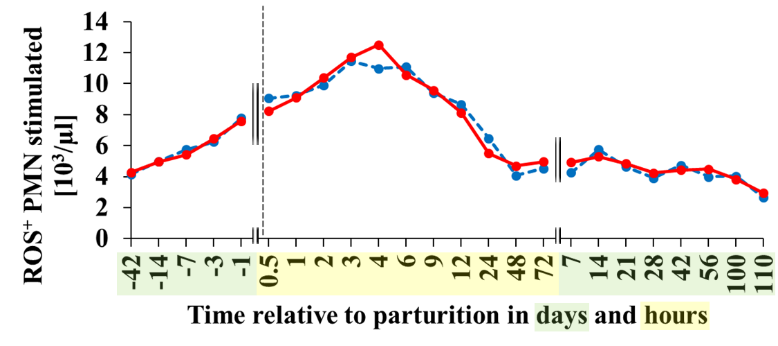 |
| (C)  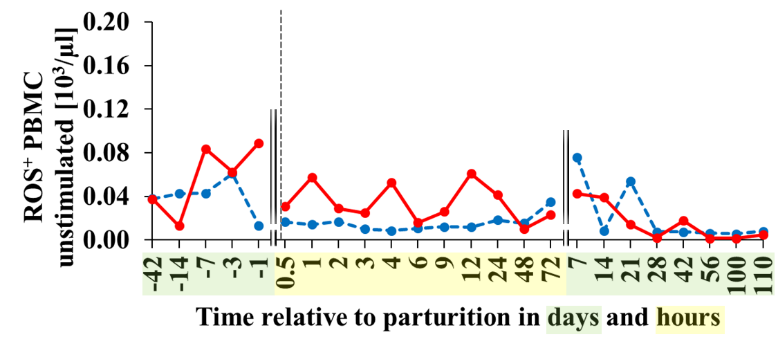 | (D)  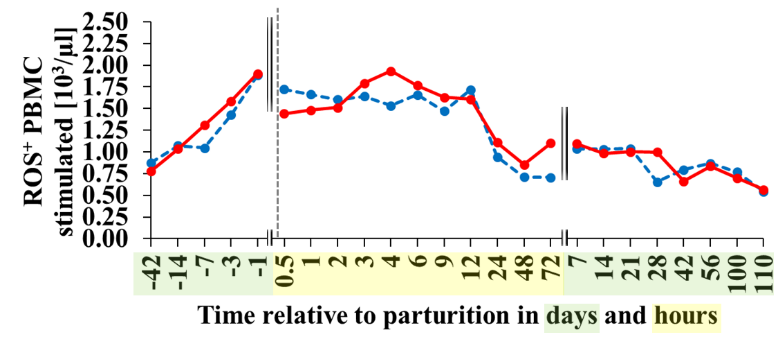 |
| \|  \|  \| **p-values** \| \| \| \| --- \| --- \| --- \| --- \| --- \| \| **Functional properties** \| **PSE^1^** \| **Group** \| **Time** \| **Group*Time** \| \| ROS^+^ PMN unstimulated (10^3^/µl) \| 0.024 \| **0.037** \| **<0.001** \| 0.997 \| \| ROS^+^ PMN stimulated (10^3^/µl) \| 0.201 \| 0.737 \| **<0.001** \| 0.808 \| \| ROS^+^ PBMC unstimulated (10^3^/µl) \| 0.005 \| 0.737 \| **<0.001** \| 0.299 \| \| ROS^+^ PBMC stimulated (10^3^/µl) \| 0.044 \| 0.519 \| **<0.001** \| 0.310 \|   (E) | |

**Supplemental Figure 1.** Functional properties of leukocytes of dairy cows fed a diet supplemented with (CAR) or without (CON) L-carnitine from 6 weeks before until 15 weeks after calving. (A) cell count of unstimulated reactive oxygen species (ROS^+^) producing PMN and (B) cell count of TPA-stimulated ROS^+^ PMN were determined by flow cytometry. (C) cell count of unstimulated ROS^+^ producing PBMC and (D) cell count of TPA-stimulated ROS^+^ PBMC were determined by flow cytometry. (E) data statistics. Data are shown as least square means. **^1^**pooled standard error.

**Supplementary Table 3:** Effects of dietary L-carnitine on phagocytic peripheral blood mononuclear cells (PBMC) on dairy cows fed a diet supplemented with (CAR) or without (CON) L-carnitine from 6 weeks before until 15 weeks after calving.

| **Days/ Hours^1^** | **Stimulation Index ROS^+3^ PBMC (%)** | **Stimulation Index MFI^4^ of ROS^+3^ PBMC** |
| --- | --- | --- |
| -42 | 52.0 | 2.84 |
| -14 | 159.1 | 2.60 |
| -7 | 186.2 | 2.53 |
| -3 | 192.4 | 2.45 |
| -1 | 421.6 | 2.06 |
| 0.5 | 438.3 | 2.22 |
| 1 | 346.8 | 2.14 |
| 2 | 446.8 | 1.93 |
| 3 | 454.8 | 2.31 |
| 4 | 393.1 | 2.29 |
| 6 | 561.7 | 2.26 |
| 9 | 431.1 | 2.17 |
| 12 | 534.9 | 2.18 |
| 24 | 286.6 | 2.75 |
| 48 | 219.0 | 3.38 |
| 72 | 257.9 | 2.98 |
| 7 | 169.4 | 3.21 |
| 14 | 269.2 | 2.05 |
| 21 | 284.9 | 1.66 |
| 28 | 254.3 | 1.76 |
| 42 | 211.7 | 1.77 |
| 56 | 354.3 | 1.55 |
| 100 | 236.9 | 1.82 |
| 110 | 136.2 | 2.27 |
| p-Value |  |  |
| Group | 0.785 | 0.387 |
| Time | **<0.001** | **<0.001** |
| Group*Time | 0.260 | 0.868 |
| PSE^2^ | 20.941 | 0.098 |

Data are shown as least square means. ^1^ day/hours relative to parturition, ^2^ pooled standard error, ^3^ reactive oxygen species, ^4^ mean fluorescence intensity.

**Supplementary Table 4:** Effects of dietary L-carnitine on leukocyte subpopulation, measured by antibody staining by cluster of differentiation (CD), on dairy cows fed diet supplemented with (CAR) or without (CON) L-carnitine from 6 weeks before until 15 weeks after calving.

| **Days/ Hours^1^** | **CD21^+^ (%)** | **MFI^3^ of CD21^+^** | **MFI^3^ of CD4^+^** | **MFI^3^ of CD8^+^** | **Ratio CD4/CD8** |
| --- | --- | --- | --- | --- | --- |
| -42 | 24.6 | 6.49 | 8.91 | 29.3 | 2.60 |
| -14 | 23.8 | 7.22 | 7.60 | 30.1 | 2.63 |
| -7 | 22.6 | 7.01 | 6.93 | 27.2 | 2.61 |
| -3 | 24.5 | 7.20 | 7.53 | 28.1 | 2.69 |
| -1 | 22.2 | 7.32 | 7.72 | 26.0 | 2.89 |
| 0.5 | 22.4 | 7.14 | 7.50 | 26.2 | 2.52 |
| 1 | 22.1 | 7.06 | 7.50 | 26.8 | 2.42 |
| 2 | 22.0 | 7.13 | 7.49 | 27.3 | 2.77 |
| 3 | 22.5 | 6.85 | 7.52 | 25.9 | 2.64 |
| 4 | 23.1 | 7.81 | 7.68 | 26.1 | 2.60 |
| 6 | 22.6 | 6.73 | 7.49 | 27.4 | 2.65 |
| 9 | 23.8 | 6.68 | 7.47 | 28.9 | 2.58 |
| 12 | 23.2 | 6.95 | 7.36 | 27.6 | 2.50 |
| 24 | 23.5 | 6.58 | 7.41 | 29.0 | 2.57 |
| 48 | 24.2 | 5.82 | 7.12 | 28.2 | 2.63 |
| 72 | 20.7 | 5.92 | 7.05 | 26.7 | 2.70 |
| 7 | 21.5 | 7.16 | 7.11 | 28.0 | 2.53 |
| 14 | 20.9 | 7.67 | 7.69 | 27.8 | 2.92 |
| 21 | 19.2 | 7.37 | 8.38 | 29.6 | 2.63 |
| 28 | 17.6 | 6.84 | 8.46 | 25.6 | 2.49 |
| 42 | 17.6 | 6.84 | 9.01 | 25.2 | 2.46 |
| 56 | 20.6 | 6.85 | 8.91 | 26.0 | 2.18 |
| 100 | 20.2 | 7.19 | 8.85 | 26.5 | 2.09 |
| 110 | 17.9 | 6.88 | 8.67 | 27.0 | 2.40 |
| p-Value |  |  |  |  |  |
| Group | 0.823 | 0.573 | 0.269 | 0.533 | 0.615 |
| Time | **<0.001** | **<0.001** | **<0.001** | **<0.001** | **0.002** |
| Group*Time | 0.777 | 0.756 | 0.149 | 0.303 | 0.533 |
| PSE^2^ | 0.444 | 1.347 | 1.059 | 4.588 | 0.055 |

Data are shown as least square means. ^1^ day/hours relative to parturition, ^2^ pooled standard error, ^3^ mean fluorescence intensity.

| 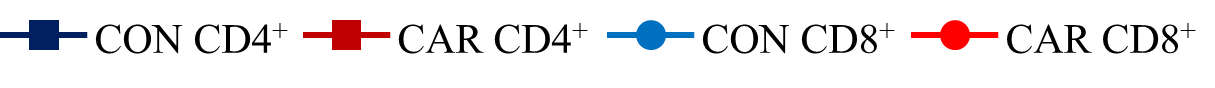 | | 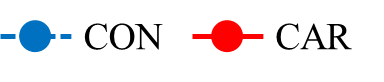 |
| --- | --- | --- |
| (A)  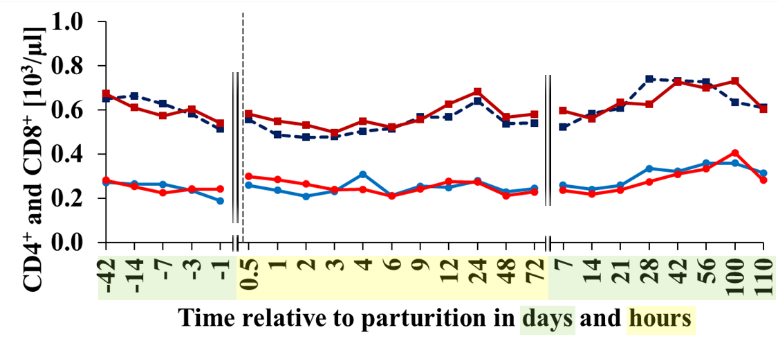 | (B)  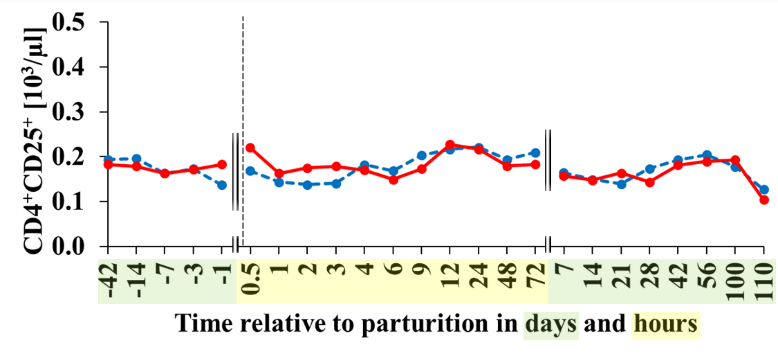 | |
| (C)  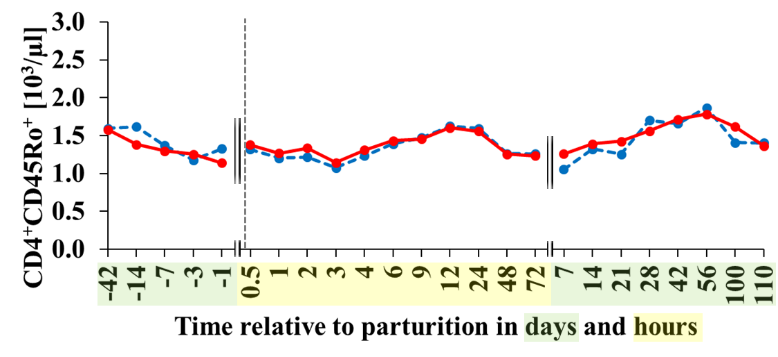 | (D)  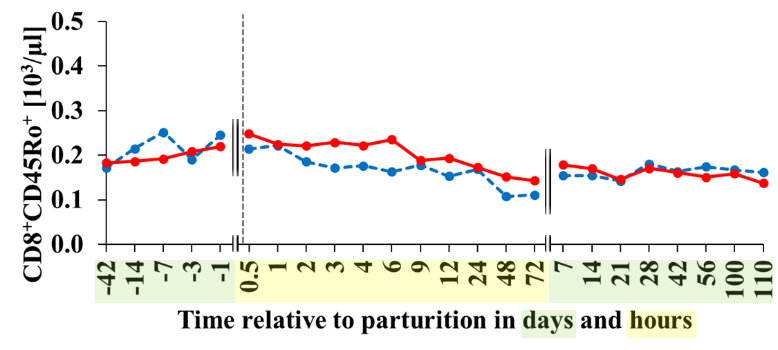 | |
| \|  \|  \| **p-values** \| \| \| \| --- \| --- \| --- \| --- \| --- \| \| **Functional properties** \| **PSE^1^** \| **Group** \| **Time** \| **Group*Time** \| \| CD4+ (10^3^/µl) \| 0.013 \| 0.379 \| **<0.001** \| 0.973 \| \| CD8+ (10^3^/µl) \| 0.009 \| 0.582 \| **<0.001** \| 0.356 \| \| CD4+CD25+ (10^3^/µl) \| 0.006 \| 0.929 \| **<0.001** \| 0.893 \| \| CD4+CD45Ro+ (10^3^/µl) \| 0.034 \| 0.757 \| **<0.001** \| 0.931 \| \| CD8+CD45Ro+ (10^3^/µl) \| 0.006 \| 0.194 \| **<0.001** \| 0.412 \|   (E) | | |

**Supplemental Figure 2.** Flow cytometric quantification of leukocyte subpopulations of dairy cows fed a diet supplemented with (CAR) or without (CON) L-carnitine from 6 weeks before until 15 weeks after parturition. Cell count of: (A) T-helper (CD4^+^) and cytotoxic T-cells (CD8^+^), (B) regulatory T-cells (Treg, CD4^+^CD25^+^), (C) memory T-helper cells (CD4^+^CD45Ro^+^), and (D) memory cytotoxic T-cells (CD8^+^CD45Ro^+^). (E) data statistics. Data are shown as least square means. ^1^pooled standard error.

| 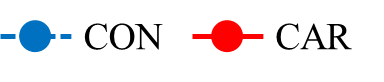 | |
| --- | --- |
| (A)  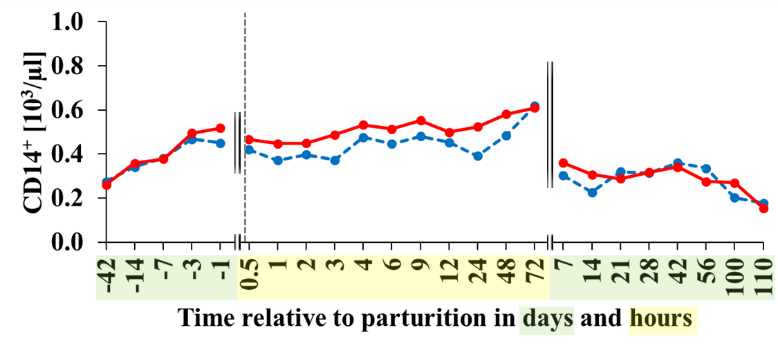 | (B)  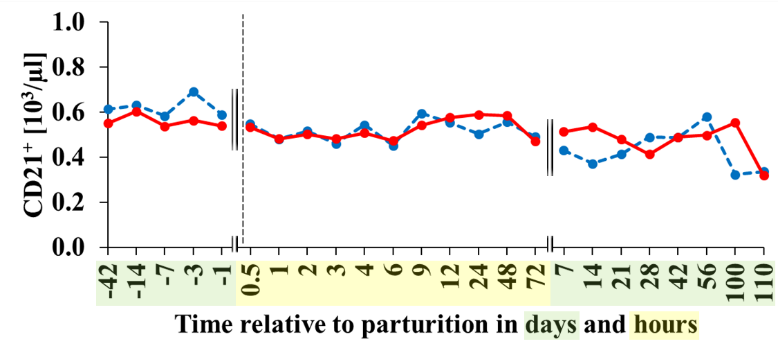 |
| \|  \|  \| **p-values** \| \| \| \| --- \| --- \| --- \| --- \| --- \| \| **Functional properties** \| **PSE^1^** \| **Group** \| **Time** \| **Group*Time** \| \| CD14+ (10^3^/µl) \| 0.013 \| **0.024** \| **<0.001** \| 0.669 \| \| CD21+ (10^3^/µl) \| 0.017 \| 0.855 \| **<0.001** \| **0.097** \|   (C) | |

**Supplemental Figure 3.** Flow cytometric quantification of leukocyte subpopulations of dairy cows fed a diet supplemented with (CAR) or without (CON) L-carnitine from 6 weeks before until 15 weeks after parturition. Cell count of: (A) monocytes (CD14^+^), (B) B-cells (CD21^+^). (C) data statistics. Data are shown as least square means. ^1^pooled standard error.
